# The Faces of “Too Late”—A Surprisingly Progressive Cohort of “Stable” Relapsing Remitting Multiple Sclerosis Patients

**DOI:** 10.3390/medicina60091401

**Published:** 2024-08-26

**Authors:** Alin Ciubotaru, Cristina Grosu, Daniel Alexa, Roxana Covali, Alexandra Maștaleru, Maria Magdalena Leon, Thomas Gabriel Schreiner, Cristina Mihaela Ghiciuc, Emanuel Matei Roman, Doina Azoicăi, Emilian Bogdan Ignat

**Affiliations:** 1 Department of Neurology, “Grigore T. Popa” University of Medicine and Pharmacy, 700115 Iași, Romania; alinciubotaru94@yahoo.com (A.C.); alexadaniel2004@yahoo.com (D.A.);; 2 Department of Neurology, Clinical Rehabilitation Hospital, 700661 Iași, Romania; 3 Department of Radiology, Biomedical Engineering Faculty, “Grigore T. Popa” University of Medicine and Pharmacy, 700115 Iași, Romania; rcovali@yahoo.com; 4 Department of Medical Specialties I, “Grigore T. Popa” University of Medicine and Pharmacy, 700115 Iași, Romania; alexandra.mastaleru@gmail.com (A.M.); leon_mariamagdalena@yahoo.com (M.M.L.); 5 Department of Morpho-Functional Sciences II—Pharmacology and Clinical Pharmacology, “Grigore T. Popa” University of Medicine and Pharmacy, 700115 Iași, Romania; c_ghiciuc@yahoo.com; 6 Department of Cardiology, Sf. Spiridon Hospital, 700111 Iasi, Romania; emy_roman96@yahoo.ro; 7 Department of Preventive Medicine and Interdisciplinarity, “Grigore T. Popa” University of Medicine and Pharmacy, 700115 Iași, Romania; doina.azoicai@gmail.com

**Keywords:** multiple sclerosis, PIRA, neurofilament light chains, EDSS, 9HPT, 25FWT, SDMT

## Abstract

*Background and Objectives*: Although available therapies have changed the natural evolution of multiple sclerosis (MS), in time some patients assume a progressive course and no longer respond to treatment. There is no definitive clinical or laboratory parameter to certify MS progression from relapsing remitting MS (RRMS) to secondary progressive MS (SPMS) in early phases of transition. Our study aims to evaluate the value of clinical parameters and serum neurofilament light chain levels (sNfLs) as early warning signs of conversion to SPMS. *Materials and Methods*: The Expanded Disability Status Scale (EDSS), Nine-Hole Peg Test (9HPT), 25-foot walk test (25FWT) and Symbol Digit Modalities Test (SDMT) were evaluated at 12 months apart in a cohort of 83 RRMS treated patients. sNfLs were evaluated at the second time point. *Results:* sNfLs correlate with EDSS and SDMT, with EDSS change and disease duration. Clinical parameters correlate among themselves and perform well in supporting the diagnosis of SPMS in logistic regression and ROC curves analysis. Eighty percent of the RRMS patients in our study (of which 65% are treated with high-efficacy disease-modifying drugs) showed some type of progression independent of relapses (PIRA) after 12 months, with one in five patients experiencing isolated cognitive worsening and almost two-thirds some type of motor worsening. We found no differences in terms of progression between patients treated with platform drugs versus high-efficacy drugs. *Conclusions*: An elevated level of progression independent of relapses (PIRA) was found in our cohort, with high-efficacy drugs providing no supplementary protection. As sNfL levels were correlated with the progression of EDSS (the main clinical progression marker), they may be considered potential prognostic markers, but further studies are necessary to precisely define their role in this direction. The lack of early sensitive markers for risk of progression may contribute to therapeutic delay and failure.

## 1. Introduction

Despite being one of the most frequent causes of disability in adults and despite generating a considerable research effort, multiple sclerosis (MS) remains elusive in terms of a definite cause or mechanism. Although it was considered for a long time that autoimmune processes are restricted to the central nervous system (CNS) and that intermittent inflammation is the key element, it is now demonstrated that neurodegenerative changes are associated from the very beginning of the disease [1]. Clinical phenotypes of MS include relapsing remitting MS (RRMS), secondary progressive MS (SPMS) and primary progressive MS (PPMS), loosely mirroring the different proportions of the two major pathological contributors. In secondary SPMS, localized acute injury is no longer the determinant, as widespread persistent inflammation and neurodegeneration become the main player and a progressive failure of compensatory mechanisms adds to the complexity of the picture [1]. The clinical shift from relapsing remitting to secondary progressive forms is frequently discrete and may be overlooked for long periods of time [2]. While somatic impairment is both the most disabling and the most at hand in terms of measurement (being the dominant factor in all diagnostic criteria), non-motor symptoms are often ignored and weigh less in the clinical decision. Cognitive disorders are a good example, being more severe and more frequent in the SPMS form compared to RRMS [3].

Progression independent of relapses is present and documented in some RRMS patients, and confirmed disability progression (CDP) at three or six months is the most frequent parameter used to measure it. Although CDP is present in all of the major pivot studies, most of the disease-modifying drugs manage to reduce its incidence in a significant manner. It is important to note that after the early studies that pointed out the lack of efficacy on secondary progression, most of the following research included almost exclusively naive RRMS patients. One of the notable exceptions is the CARE MS II study, where patients with active disease despite platform therapies received either alemtuzumab or interferon beta 1 b [4]. In a pooled posthoc analysis of the evolution of the patients in the two CARE-MS studies, 34% (172 out of 511 subjects) presented 6 months confirmed disability worsening anytime during the 9 years of follow-up. The mean time since first symptoms for patients with CDW was 4.0 ± 2.8 years [5]. In the AFFIRM study, the cumulative probability of progression (including both relapse-related and relapse-independent) was 17% in natalizumab-treated patients (a relative 42 percent decrease in the risk of a sustained progression of disability, hazard ratio, 0.58), with an adjusted annualized relapse rate of 0.24 (0.75 for the interferon-treated patients) [6]. In the OPERA I and OPERA II trials, 9.1% of the patients receiving ocrelizumab (versus 13.6% of those receiving interferon beta 1a, hazard ratio, 0.60) presented disability progression confirmed at 12 weeks and only 6.9% (vs. 10.5%; hazard ratio, 0.60) with disability progression confirmed at 24 weeks [7]. In the same studies, the annualized relapse rates were 0.16 in the ocrelizumab group and 0.29 in the interferon patients.

Summarizing the previous paragraph, it is expectable that 5–15% of treated RRMS patients with active disease would present relapse-independent disability progression while achieving a nearly complete suppression of inflammatory activity. These numbers are expected to be nearly halved if HETs are used. In patients with unsatisfactory response to treatment, numbers are slightly higher (as shown by the CARE-MS II study where 40 (20%) patients in the interferon beta 1a group had sustained accumulation of disability over 2 years, compared with 54 (13%) in the alemtuzumab group (hazard ratio 0.58), corresponding to a 42% improvement in the alemtuzumab group) [4].

Proportions are different in secondary progressive patients. In ASCEND, the capacity of natalizumab to slow disease progression in SPMS patients was evaluated in comparison to placebo. Subjects were known with SPMS for at least two years and presented disability progression independent of relapses in the previous year. In the first part of the study, 44% of 439 natalizumab-treated patients and 48% of 448 placebo-treated patients had confirmed disability progression [8]. Of all the tested parameters, natalizumab had a significant favorable influence only on the impairment of the upper extremity.

Although the available disease-modifying therapies (DMTs) do not show satisfactory efficiency in progressive/neurodegenerative aspects of MS, there are data suggesting that high-efficacy therapies (HETs) could slow down clinical worsening even when used during the secondary progressive phase [9]. Switching patients from platform therapies to HETs when progression is suspected could, therefore, be useful, but we can assume that early intervention would be a key determinant to reach the desired outcome. At present, no test or biomarker can establish with certainty the initial moment of secondary progression in MS. The diagnosis is mostly retrospective, based on the corroboration of clinical and history data [10,11,12,13], and the uncertainty usually lasts for about three years [2]. A clear delimitation of a “pre-SPMS” or “very early SPMS” phase would define a window of intervention where the effect of HETs on progression could be evaluated, and eventually lead to a clear indication to escalate treatment.

Although the search for a reliable MS biomarker produced mostly disappointing results in the past century, during the last decades a few promising tests have emerged (for a quick review of MS biomarkers see [14]). Of these, the serum level of neurofilament light chains (sNfLs) appears to be the closest to widespread clinical use. Neurofilaments are components of the cytoskeleton that are released upon neuronal injury, and make their way into the serum, plasma and cerebrospinal fluid from lesioned axons [15]. Both the sensitivity and specificity of this biomarker depend on the laboratory technique and on the associated conditions, and they are not specific to MS [16]. Although sNfLs have proven their utility mainly as markers of disease activity and to monitor therapeutic efficiency [15,17,18], there are data supporting their use for predictive purposes regarding the progression of the disease in the short and long term [17,19,20].

While in humans the value of sNfL and CSF NfL levels are progressively gaining ground as a disease activity prognostic biomarker, their value in preclinical models of MS is still unclear. While neurofilaments, particularly neurofilament light chains (NfLs), are important biomarkers for neurodegeneration in MS, their dynamics in animal models may not accurately reflect those in human patients. This discrepancy can hinder the assessment of neurodegeneration and treatment efficacy.

Animal models, particularly for experimental autoimmune encephalomyelitis (EAE), are essential for studying multiple sclerosis. However, they come with significant limitations that impact the translation of findings to human disease—many treatments that show promise in animal models do not translate effectively to results of human clinical trials. This is partly due to the differences in disease mechanisms and the complexity of MS, which cannot be fully replicated in animal models. No single animal model captures the full spectrum of MS pathology, including its clinical, immunological and pathological features. EAE mainly reflects the autoimmune aspects of MS but does not adequately represent the contributions of other immune cells, such as CD8+ T cells and B cells, which are crucial in the progressive stages of MS. Current models provide insufficient information about the mechanisms underlying progressive MS, particularly regarding neurodegeneration and axonal injury, which are critical for understanding disease progression and developing effective therapies.

In a recent meta-analysis on the translational value of NfLs in the experimental autoimmune encephalomyelitis (EAE) mouse model, the authors evaluated twenty-five studies on this model, and concluded that there was a highly significant correlation between plasma and cerebrospinal NfL levels and the EAE clinical scores, confirming that NfLs are a robust predictor in the EAE mouse model [21].

This study is part of an ongoing prospective attempt to reevaluate our aging cohort of treated MS patients and our current practices. In the longer term, we aim to validate the clinical diagnostic criteria for SPMS that were proposed by a workgroup of the Romanian Society of Neurology (unpublished) and to find an approach that suits patients who, in the absence of classical indications for treatment escalation, present with signs that suggest an unsatisfactory disease control, allowing for a timely suspicion of clinical disease activity not associated with relapses. sNfLs were evaluated in seemingly stable patients, in an attempt to establish correlations with discrete progression notrelated to relapses. At present, evaluation of sNfLs is not a part of the standard monitoring of MS patients in our center.

## 2. Materials and Methods

The study was conducted between January 2022 and December 2023 on a cohort of 83 patients with RRMS (2017 McDonald criteria) treated in the Neurology Clinic of the Clinical Rehabilitation Hospital in Iași, Romania. According to the McDonald criteria, a patient is diagnosed with MS after an initial clinical suggestive episode, exclusion of other possible etiologies and fulfillment of the dissemination in time and in space criteria [22].

From the 150 patients for whom we had clinical data recorded one year before, we have selected the first 83 patients who met the criteria (as limited by available funding). Patients signed an informed consent before sNfLs were tested. Historical data (including the results of clinical tests) wereextracted from patients’ files. 

We selected patients with a confirmed diagnosis of RRMS according to the 2017 McDonald criteria, a disease duration longer than three years at the first evaluation, aged over 18 years at inclusion. Exclusion criteria were: refusal to participate in the study; psychiatric conditions that affect ability to agree to study participation; any type of orthopedic/peripheral damage that would prevent completion of the tests; treatment with corticosteroids or relapse in the past 18 months (6 months before the initial evaluation and 12 months between the two time points). 

From the initial group, 29 patients transferred, did not come to visit in due time or did not meet the inclusion/exclusion criteria. Three patients who relapsed between the two time points were excluded. One patient refused to participate. 

All patients received disease-modifying therapies as prescribed by their treating neurologists. Therapy was coded into high-efficacy therapy (including natalizumab, ocrelizumab, cladribine, alemtuzumab and fingolimod) and platform therapy (including beta interferons, glatiramer acetate, dimethyl fumarate).

Clinical and history parameters were recorded at two time points (T0 and T1) at 12 months distance, between 2022 and 2023. sNfLs were evaluated only at the second timepoint (T1). 

Demographic/history variables were sex, age, disease duration, treatment type, current treatment duration, reason for treatment escalation for patients on high-efficacy therapy (treatment failure, highly active MS at onset, increased risk of PML, adverse events).

Clinical parameters were the Expanded Disability Status Scale (EDSS) (global disability), 9-Hole Peg Test (9HPT) (upper limb function); 25-Foot Walk Test (25FWT) (gait/lower limb function); Single Digit Modalities Test (SDMT) (cognition). The 25FWT and 9HPT were performed as stated in the Multiple Sclerosis Functional Composite Measure manual [23]. The oral form of the SDMT was applied [24]. For the 9HPT, we used a standard Jamar 9-Hole Peg Test kit. For SDMT, we used a variant of the original evaluation sheet. A standard chronometer watch was used for all the tests. 

EDSS change was calculated by subtracting EDSS at T0 from EDSS at T1. For 25FWT and 9HPT, average values of the tworepetitions of each test were used and the percentage change in both scores was calculated by relating the difference between the scores (at T1 versus T0) to the initial value (at T0).

The criteria proposed by the Romanian Society of Neurology for the diagnosis of MS were used to define “events” for the EDSS, 25FWT, 9HPT and SDMT scores. An EDSS “event” was defined if the EDSS increased more than 1 point starting from an initial EDSS below 5 and with more than 0.5 points starting from an initial EDSS of more than 5.5. A 25FWT or a 9HPT “event” was defined if average times increased with more than 20% from the initial values. Due to the possibility of unilateral motor weakness in MS, in the analysis of 9HPT results we have used the maximal percentage of change in either limb (largest percent from the dominant and non-dominant upper limb). Consequently, to record a 9HPT event a 20% increase had to be present in at least one of the upper limbs. AnSDMT “event” was defined by a decrease of at least 4 points compared to the T0 evaluation. As the criteria state that progression in the absence of relapses, measured for one year retrospectively or 6 months prospectively, is verified by at least one of the EDSS, 9HPT or 25FWT “events”, with SDMT and age as accessory criteria, we defined an “SPMS event” (equivalent to the diagnosis of SPMS) in patients with at least one of the EDSS, 25FWT or 9HPT events.

The dosing of sNfLs was carried out by an authorized private laboratory using the single molecule array (SIMOA) technique on a Quanterix SR-X/SIMOA HD-1 apparatus (Quanterix, Billerica, MA, USA). sNfL levels are presented in pg/mL and normal values were adjusted for age and sex.

### 2.1. Ethical Consent

The present study was approved by the ethics committees of Iași Clinical Rehabilitation Hospital and “Grigore T. Popa” University of Medicine and Pharmacy Iași (22/16 November 2022; 265/1 February 2023). All the interventions were carried out in accordance with their regulations and those of the Declaration of Helsinki.

### 2.2. Statistical Analysis

Data analysis was performed using SPSS 26.0 (Statistical Package for the Social Sciences, Chicago, IL, USA). Data were presented as mean ± standard deviation for continuous variables following a normal distribution, as median with interquartile range (Q1, Q3) for variables not conforming to a normal distribution and as frequency and percentage for categorical variables. Categorical variables were compared using the Chi-square test. The relationship between various variables was assessed using the Spearman correlation test (with few exceptions data did not respect a normal distribution). Binomial logistical regression was used to estimate the capacity of various patient-related parameters to predict the probability of an SPMS event. The independent samples Kruskal–Wallis test, independent samples Mann–Whitney U test and Wilcoxon signed rank test were used to compare non-normally distributed data. Receiver operating characteristic (ROC) curve analysis was performed, and areas under the curve (AUCs) were calculated to estimate the predictive performance of patient variables. Statistical significance was determined by a *p*-value < 0.05 for all statistical tests.

## 3. Results

We evaluated 83 clinically stable Caucasian patients with RRMS. Average age at T0 was 41 ± 12.039 years (18–66 years). About two-thirds were female (48, 57.84%). Mediandisease duration was 10 years (6, 16), ranging from 3 to 34 years. All patients had started disease-modifying therapies at least 6 months before T0 and continued it through the duration of the study. Median duration of the current therapy was 2 years (1, 5), ranging from less than a year to 20 years. High-efficacy treatment was used in more than half of the patients (52 patients, 62.7%), with natalizumab (the most frequent) in 29 patients (34.9%) followed by ocrelizumab in 17 patients (20.5%) and immune reconstitution therapies (cladribine and alemtuzumab) in five patients (6%). The rest of 31 patients (37.3%) were treated with platform drugs (beta interferons in 17 patients representing 20.4% from the entire group). The reasons for introducing high-efficacy treatment were: insufficient disease control in 42 patients (80.8% of the HET-treated group), highly active disease at the debut in seven patients (13.5%) and concerns regarding progressive multifocal leukoencephalopathy in three patients (5.8%). In the ocrelizumab-treated group, 35% (six patients) were treatment-naive or came from natalizumab because of increased PML risk, while in the natalizumab-treated group only 10.3% (three patients) were treatment-naive, with the rest failing at least one other therapeutic course before.

Insufficient disease control (or persistent disease activity despite disease-modifying treatment) and escalation to high-efficacy treatment require, according to local guidelines, the presence of at least a relapse during the past year (with or without proof of MRI activity) or proof of disease activity at MRI at repeated evaluations (usually 6 months apart).

Treatment was stable along the duration of the study.

Median values for all the target parameters are visible in Table 1. The variation of scores between T1 and T0 appears in the last column, either as a difference (EDSS, SDMT) or as a percent of change (9HPT, 25FWT).

Final (T1) EDSS was significantly higher than initial EDSS (Z = −4.357, *p*< 0.001). EDSS increased on average by 0.265 points, as EDSS changes ranged from a 0.5 decrease to a 2-point increase. We found EDSS increases in 25 patients (30.12%) but only in 20 patients (24.09%) did these qualify as EDSS events. One patient (1.2%) presented a 0.5-point reduction in the EDSS and the rest were stable. 

EDSS correlated well with 9HPT, 25FWT, SDMT both at T0 and at T1 (see Supplementary File S1 for all correlation coefficients and significations). However, it did not correlate (neither as static values nor as difference) with 9HPT and SDMT worsening (differences). Gait worsening was related to initial and final EDSS (rho = 0.361 and 0.363, respectively, *p* = 0.001 for the correlation between 25FWT difference and EDSS at T0 and T1), but not to EDSS worsening (no correlation between EDSS difference and 25FWT difference).

The 9HPT times were relatively stable, with no significant difference between T1 and T0 in either limb (*p* = 0.548 for the dominant UL and *p* = 0.136 for the non-dominant UL); the Wilcoxon Signed Rank Test did not show significant differences between upper limbs (dominant versus non-dominant) at T0 (Z = −1744, *p* = 0.081), as opposed to T1 (Z = −2730, *p* = 0.006). Although the median percentage of change was different in the two upper limbs, this did not reach statistical significance (Z = −0.831, *p* = 0.406). A 9HPT event (maximal change above 20%) was registered in 22 patients (26.5%). 

Gait speed decreased as median 25FWT time increased significantly during the study, Z = −3.389, *p* = 0.001. A 25FWT event was registered in 22 patients (26.5%). 

SDMT scores significantly decreased (Z = −6.694, *p*< 0.001). An SDMT event was registered in 48 patients (57.8%). Higher SDMT scores at both time points correlated with the use of high-efficacy treatment (rho = 0.284, *p* = 0.009 at T0 and rho = 0.270, *p* = 0.014 at T1). SDMT difference correlated negatively with upper limb dexterity—9HPT worst upper limb score (rho = −0.237, *p* = 0.031).

sNfL levels were normal in the majority of cases with only two patients having values above the sex- and age-adjusted normal.

**Figure 1:**
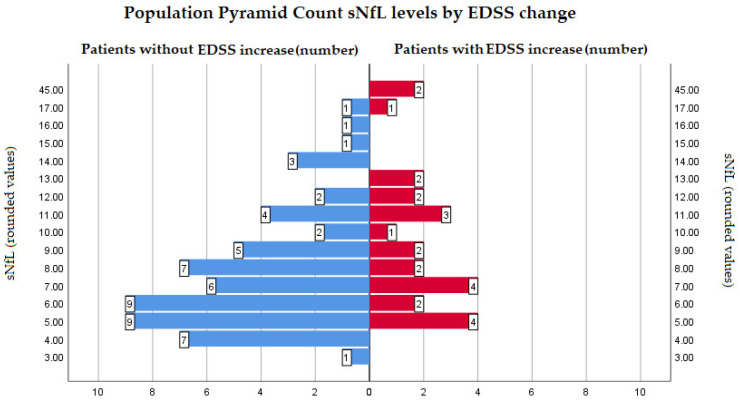
Population pyramid count of sNfL values in relation to presence of EDSS change at T1 compared to T0 (Bars represent number of patients with a specific sNfL value). Values were rounded to the nearest integer for this graphic. Mean ranks for patients without EDSS change were 38.34 (N = 58) and for patients with EDSS change 50.5 (N = 25)—Mann–Whitney U test was significant *p* = 0.035. Abbreviations: sNfL: serum neurofilament light chain; EDSS: Expanded Disability Status Scale.

sNfL levels correlated with EDSS change (rho = 0.255, *p* = 0.02) and with disease duration (rho = 0.394, *p*< 0.001). sNfL levels also correlated well with the EDSS score at the moment of the test (T1)—rho = 0.411, *p*< 0.001 and with the SDMT score at T1 (rho = −257, *p* = 0.19), as well as with the same parameters at T0. The distribution of sNfL levels in relation to presence or absence of EDSS change is visible in Figure 1.

sNfL was the only significant determinant (beta = 0.022, sig = 0.004) in a linear regression model with EDSS difference as dependent and the initial values of clinical measures, age, disease duration, treatment type and sNfL levels as predictors (overall model significance was 0.012, adjusted R square 0.149). The relation between sNfL and EDSS difference is maintained with any combination of predictors.

At the end of the follow-up period, 49 (58.33%) of the patients fulfilled the criteria for an SPMS event. 

A binary logistic regression model including age, disease duration, treatment duration, treatment type and the initial values of EDSS, 9HPT, 25FWT and SDMT as determinants for an SPMS event did not show significant influences of any of the above, despite the overall model being significant (omnibus test *p* = 0.024, Hosmer and Lemeshow goodness of fit test *p* = 0.687)

Differences recorded in all of the motor parameters correlated with SPMS events—25FWT time difference (rho = 0.454, *p* < 0.001), 9HPT worst upper limb score difference—(rho = 0.376, *p* < 0.001) and EDSS difference (rho = 0.307, *p* = 0.005). The same correlations were present when events were used instead of the discrete difference values, with slightly higher correlation indices.

We have used logistical regression to evaluate the effects that initial EDSS score, changes in EDSS, 9HPT, 25FWT and SDMT, disease duration, treatment type and sNfL levels have on the likelihood of a patient to present an SPMS event/fulfill the criteria for SPMS. Although the logistical regression model was significant (chi square = 55.083, *p* < 0.001), only motor performance tests worsening had a significant association with conversion to SPMS, with a 2.687 increase in the odds to develop SPMS for each one-point increase inthe EDSS (increases in9HPT and 25FWT completion times had a more modest impact). The model explains 68.1% of the variance of SPMS conversion (NagelkerkeR2) and classifies correctly 79.5% of the cases. 

We used ROC curves to evaluate the performance of various clinical and laboratory measures in classifying MS patients into SPMS (SPMS event present) versus RRMS (SPMS event absent) categories. Curves for static parameters (initial EDSS score, disease duration, sNfL level) and for measures of change during the 12 month follow-up (EDSS score differences, percentual changes of the 9HPT for the worst upper limb, percentual changes of the 25FWT completion time and the SDMT score differences) were calculated. Areas under the curve for 25FWT difference and for 9HPT highest difference are above 0.7 (statistically significant in both cases). Still significant, but with AUC between 0.6 and 0.7, are EDSS at T0, EDSS difference and disease duration. SDMT difference and sNfL levels are near 0.5. Cut-off values for the measured parameters closest to those used in the diagnostic criteria and the corresponding sensitivity and 1-specificity are presented in Table 2.

**Figure 2:**
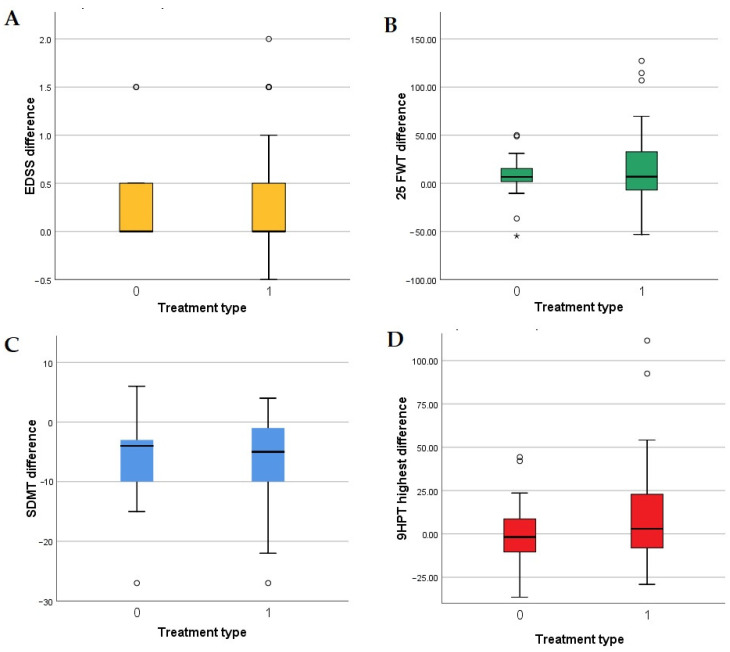
Distribution of EDSS differences: (**A**), 25FWT differences (**B**), SDMT differences (**C**) and 9HPT differences (**D**) across treatment type (0 represents platform treatment; 1 represents high-efficacy treatment). Abbreviations: 9HPT: 9 Hole Peg Test, 25FWT: 25 Foot Walk Test; EDSS: Expanded Disability Status Scale; SDMT: Single Digit Modalities Test.

Since we expected patients that are treated with HETs to have a more stable evolution, we have compared the variation of the clinical parameters over the duration of the study in relation to the presence or absence of HETs. We found no significant differences in terms of distribution of EDSS difference, 25FWT difference, SDMT difference and 9HPT difference in the two treatment-defined categories (independent samples Kruskal–Wallis test significances were 0.649, 0.824, 0.882 and 0.169, respectively). Distributions of each of the parameter variations in relation to treatment are represented in Figure 2.

## 4. Discussion

Disease-modifying therapies—platform drugs and HETs—have changed the livesof most MS patients. Still, there are some who appear to be out of reach for the available drugs’ resources. In close relation to this, the diagnosis of SPMS is decisive for the patient:labeling a patient with this diagnosis dramatically narrows therapeutic options and perspectives, and can be perceived by both the patient and the neurologist as abandonment. With the exception of siponimod, subcutaneous interferon beta 1 a (both indicated for SPMS with activity), mitoxantrone and interferon beta 1 b (all with modest results), no other substance is indicated for the treatment of SPMS, directly reflecting the lack of proven efficacy. Acknowledging that some of our RRMS patients may in fact already have changed to SPMS is therefore not a surprise, since in some cases we willingly maintain or switch treatment to HETs to avoid giving up. The main disadvantage is the exposure to unnecessary risks in the absence of proven benefit. Since studies of disease-modifying therapies yielded negative or at most unclear results in terms of progression independent of relapse activity (PIRA) (reviewed in [25]), switching patients to a higher efficacy drug is useless in theory. However, there are data that support treatment escalation in SPMS patients—most recently in [9] the authors found that the proportion of treated SPMS patients who developed PIRA at 48 months was significantly higher in IFNb-1b-treated compared to natalizumab-treated patients (72.4% versus 40.2%, *p* = 0.01), with IFNb-1b patients being 1.64 times more likely to progress (HR 1.64, 25%CI 1.04–4.87; *p* = 0.001). 

In contrast with our findings (with similar rates of progression in natalizumab and platform drug-treated patients), in a recent systematic meta-analysis including 27 studies with highly active and sub-optimally treated RRMS patients the authors find that in terms of disability, patients receiving natalizumab had a significantly lower rate of 3-month confirmed disability progression at 50-month follow-up compared with platform drugs. However, for all other CDP outcomes and time-points (12- and 24- month follow-up), there was no significant difference for natalizumab-treated compared with platform DMT-treated patients in the main, or sensitivity, analysis. Compared to fingolimod, natalizumab-treated patients had significantly lower 6-month CDP at 48-months follow-up but there was no significant difference for other CDP outcomes and time-points (12 and 24 months). In a case series, the proportion of natalizumab-treated patients with 3- or 6-month CDP was 0% and 2.6%, respectively, at 1- to 2-year follow-up [26]. 

In another retrospective study including patients that have received natalizumab for at least 2 years [27], the authors conclude that almost 80% of them did not develop PIRA and remained stable despite a mean disease duration of more than 15 years. However, in the 20% of the patients that did develop PIRA the EDSS increased under natalizumab with a mean change of 1.4 ± 0.9 compared to baseline. When comparing the results with existing natural disease course data, the authors found that the rate for conversion to a secondary progressive disease course may be reduced by 50% under natalizumab. As opposed to the majority of studies that separate patients with EDSS below and above the 5–5.5 area, in this study authors considered the phase of moderate disability with EDSS 3.5–5.5 to be particularly relevant for PIRA development and therefore chose to increase the sensitivity for change in this range. However, confirmed PIRA risk was not different between the EDSS < 3 and >3.5 group [27].

In a post-marketing study, disability worsening-free survival in ocrelizumab-treated patients was 90.5% and 68.8% for RRMS and SPMS patients, respectively [28]. In another recent retrospective study, 86% of the ocrelizumab-treated patients were progression-free in the first year of treatment, 71% in the second year, 64% in the third year and 62% in the fourth year. During the follow-up of the cases, EDSS worsened in 9% of RRMS patients and in 40% of patients with SPMS with relapses [29]. Lower rates of progression were found in a shorter Spanish study (around one year), with 3.3% (two out of 60 patients) of the RRMS patients progressing and 17.6% (three out of 17) of the SPMS patients progressing [30]. In another retrospective study [31], the authors conclude that, in ocrelizumab-treated RRMS patients, the main driver of disability accumulation is PIRA rather than relapse-associated worsening. During the 29 months mean follow-up time, 23.5% of the 79 patients developed confirmed disability accumulation under ocrelizumab therapy, with the majority of those developing PIRA (87.0% of CDA, *n* = 20) rather than relapse-associated worsening (RAW) (13.0% of CDA, *n* = 3). In a rather unexpected finding, the two possible factors associated with an increased probability of developing PIRA were a shorter disease duration prior to ocrelizumab (*p* = 0.02) and a lower number of previous DMTs prior to ocrelizumab (*p* = 0.04) [31].

A systematic review (including only one of the studies above) finds that CDP in RRMS patients was reported in 12 studies, with patient numbers ranging from fiveto 946: 11 studies reported CDP in fewer than 10% of patients (ranging from 0% to 9.5%) and one study reported CDP in 20% of patients at a mean follow-up of 5.6 months [32]. The majority of studies mentioned above use only EDSS as a marker of progression. In addition, the relatively small differences in threshold definition (the EDSS level where smaller changes have a more important impact) span the 3.5–5.5 interval (thus covering most of the moderate disability and reducing the reliability of the comparison). Choosing the 5.5 threshold increases the specificity of the SPMS diagnosis while choosing 3.5 increases the sensitivity. Adding more parameters could provide a better level of sensitivity in this area (as an example, the degree of impairment that allows a patient to walk between 100 m and 200 m, corresponding to a 0.5 increase in the EDSS, covers a wide area of individualized disability). Various parameters—such as disease duration, number of disease-modifying therapies, disease activity before the current drug—can induce a significant bias.

Although the median disease duration for the patients in our study group is 10 years, median duration of current treatment (DCT) is 2 years, reflecting relatively recent therapeutic changes. The correlation between treatment type (HET) and a DCT of less than 3 years (rho = 0.354, *p* = 0.001) also reflects the fact that high-efficacy treatment was started recently in many of our patients. As shown by the high rates of worsening independent of relapse that we found, it is possible that, at least in some of them, delayed therapeutic switch allowed the degenerative mechanisms (mirrored by the progressive course of the disease and PIRA) to reach a critical point. We would have expected HET to be associated with clinical stability. Since tests comparing differences of motor and cognitive parameters in relation to type of treatment or to treatment duration did not highlight statistically significant differences (independent samples Kruskal–Wallis test) (Figure 2, Supplementary File S2), at this time our conclusion is that HET had no supplementary protective effect in non-relapsing patients in our cohort. A cohort-related effect is possible, with patients on platform drugs in our group being more stable in general (not having required an escalation of the therapy), and patients recently changed to HET being less well-controlled during the years before our study, hence more prone to progress. 

In our study, sNfL levels correlated with the increase in the EDSS score, and sNfLs were significantly higher in patients that had experienced disability progression (EDSS increase) during the follow-up period. However, there were no correlations with the other progression markers or with the SPMS event. Due to the fact that sNfL levels were measured only at the second visit, we cannot speculate on a possible prognostic value. Our findings are supported by the results of a prospective study that followed a large group of patients for 12 years [33]. In this study, sNfL levels were significantly associated with EDSS at baseline and along the evolution and significantly increased in relation to EDSS worsening. However, initial levels of sNfL were not prognostic for future EDSS worsening. Although the cited study was not designed to evaluate the effect of therapy on sNfL levels, when the evolution is analyzed in terms of treatment types the results show a sustained effect of HET, reflected in different levels of sNfL at baseline depending on the type of therapy and greater reduction in sNfL levels over time in patients starting on HET or (more pronounced) switched to HET from platform therapies during the study [33]. 

At the end of the 12 months follow-up period, in the absence of relapses, 66 out of the 83 patients (79.51%) were found to have worse performance in at least one of the tests we used. Of these, 17 (20.43%) presented only cognitive decline and 49 (58.33%) motor disability progression. Only one patient declined in all four items (EDSS, 9HPT, 25FWT, SDMT) and 12 (14.45%) in three out of the four. Although the test results correlate well among themselves at both time points (including the SDMT), their changes at 12 months do not. As the only exception, we found a weak negative correlation (rho = −0.237, *p* = 0.031) between upper limb disability increase (9HPT difference) and cognition decrease (SDMT difference). Since we have used the oral (and not the written) version of the SDMT, we consider that the cognitive worsening is true and not related to a physical problem preventing the patient from efficiently completing the test. We also have to point out the fact that the distance between time points and the low number of the evaluations does not allow us to be sure whether the said disability progression is truly persistent or whether it is relatively recent and subject to possible remission. Most of the diagnostic algorithms/criteria proposed for SPMS heavily rely on motor performance—in most cases EDSS increase is critical, with baseline EDSS, motor function sub score, 25FWT, 9HPT being the other required tests in some cases [13,34] and patient age assuming a determinant role in others [35,36]. Although cognitive evaluation is recommended, cognitive loss is most of the times “suggestive” for progression but not part of the decision algorithms.

If we consider the two stages of MS as a continuum, with neurodegeneration/progression slowly taking over in time, we could interpret its clinical manifestation as a sign of treatment failure and need for more aggressive therapy. The threshold we set for this is critical, as late intervention probably carries less benefit. An increased sensitivity and specificity of the diagnostic of progression (even before one can call it secondary progressive MS) is needed for all patients, but especially for those at in an early progressive phase of the disease when there is still potential for efficient interventions. 

Serum NfL levels are a promising marker for active RRMS, but also for progression. In [37], the authors find that progressive MS patients have higher values of sNfL, and that baseline sNfL levels were associated with future longitudinal atrophy of the grey matter. In [20], the same group conclude that an increased level of sNfL is associated with a worse prognosis of cognitive function (r= −0.265), gait speed (r = 0.235), manual dexterity (r = 0.337). While in their study sNfL levels were different in relation to cognitive status and related to evolution, in our patients we did not find significant differences in sNfL levels in patients with a normal SDMT at T0 versus abnormal SDMT at T0, or in patients with SDMD decrease as compared with the cognitively stable (independent samples Kruskal–Wallis test, Supplementary File S2). 

It is important to note that in our patients SDMT scores at T0 were more than 2SD below normal according to normative data of the test [24] in 63 patients (75.9%) (median SDMT at T0 in our study was 32 (20, 41) while normative data according to sex and age generated a median of 42 (42, 43)). In our study, SDMT decrease (expressing cognitive loss) was not significantly different in cognitively normal as compared to cognitively impaired subjects (Kruskal–Wallis test, Supplementary File S2). In another study, cognitive dysfunction, measured using BICAMS, was found to correlate with raised CSF NfL levels in only the progressive forms of MS [38]. We also found correlations between sNfL levels and cognition both at T0 and at T1 (SDMT–sNfL correlation was significant with rho = −0.255 at T0 and rho = −0.257 at T1). Similar results came from a more in-depth study [39], where the authors find that while NfL levels from serum and CSF correlate with cognition at baseline and at 10 years, they were not associated with the rate of SDMT decline as shown by longitudinal analyses. 

Both the T0 and T1 values and the T1–T0 changes of EDSS, SDMT and 25FWT correlated with conversion to SPMS, and had a significant impact on this diagnosis in the regression model, as expected. The existing correlation between upper limb ability on one side and EDSS and gait speed on the other shows that it was severe enough to change EDSS (when half of the patients had an EDSS above 4 and a fourth above 5.5, showing gait impairment). Both 9HPT and 25FWT median times in our patients are well above normal values [40,41].

In a prospective cohort study with a clinical follow-up of more than 15 years, Thebault et al. evaluated the prognostic role of baseline NfL levels [29]. In terms of progressive disease, while finding a trend for higher median levels of sNfL in patients with progressive forms (sNfL  =  11.76  ±  7.88 pg/mL for progressive patients and sNfL  =  9.08  ±  7.80 pg/mL for remitting patients, *p* = 0.082), they strongly support sNfL utility as prognostic markers for progression. AUC for conversion to SPMS was 0.744 (95% CI 0.61–0.88, *p*  =  0.054), and they conclude that a sNfL value of 7.62 pg/mL was the best cut-off point to predict a course of MS progression in long-term follow-up, with patients with levels above this value being 4.3 times more likely to reach an EDSS of 4 and 7.1 times more likely to develop progressive MS [42]. In contrast with their findings, in our patients the median sNfL was 7.49 pg/mL (5.44, 10.5), similar to that in the control group in the cited study. Replacing the laboratory-provided normal values with 7.49 pg/mL resulted in 39 (46.98%) of our subjects having “abnormal” values. Repeating the analysis in this context did not bring out different results, with one exception—when conducting univariate analysis of variance (ANOVA) to assess the impact of sNfL levels on change in motor and cognitive parameters we found significant differences in terms of EDSS change in subjects with values of the sNfL higher than 7.49 pg/mL (F = 4.455, *p* = 0.038), explaining 4% of the variance of EDSS change (R^2^ = 0.052, adjusted R^2^ = 0.04). 

In a similar manner to the other parameters, disease duration correlated with all items both at T0 and T1 points, but did not correlate with any of the changes that were recorded at 12 months. It did not have a significant contribution to SPMS diagnostics in the logistic regression model but generated an AUC over 0.6 in the ROC curve analysis. 

The model of diagnostic criteria we have used in this study needs further validation and would probably benefit from the inclusion of cognitive tests among the criteria. Although a marker that precedes clinical thresholds would open new possibilities for better therapeutic approaches, for now our data cannot support the use of sNfL in this direction. As opposed to normal values, defining a lower alarm threshold (similar to that proposed by Thebault [42]) might be a more sensitive marker for progression risk. The literature is conflicted on their usefulness in progressive forms of MS, but there are studies that strongly support it. We cannot state whether the results we had are due to inherent characteristics of NfL release in relation to pathogenic mechanisms, are a result of an array of external factors related to our patient group or are mostly related to the design of our study. Although the immediate effect of treatment on increased NfL levels associated with activity is well-documented [43], long-term follow-up studies and studies in treated non-relapsing patients are missing. Careful study design is needed in order to mitigate the possible confounding factors. While reviewing the literature, we found that most studies that provide a good longitudinal follow-up have limited numbers of subjects, making them sensitive to influences that cannot always be anticipated.

Lack of impact of HETs as compared to platform therapies could be related to the mode of action of those drugs. In our study, the similar impact of ocrelizumab and natalizumab (with completely different mechanisms) is probably due to the characteristics of our group. Although more of the patients on natalizumab were “suboptimal therapy” patients, they behaved in a similar manner tothe ocrelizumab group (including more recent and more stable patients at inclusion). Number of previous therapies and level of disease activity before switching to HET should also be taken into account. In [27], the authors find that patients that initiate natalizumab earlier in the disease seem to be more likely to develop PIRA than those with late initiation, independent of natalizumab treatment duration. Although this seems contrary to our own view that earlier treatment should decrease the risk of progression, the authors of the abovementioned paper relate this to a more severe disease at the beginning that increases the need for HETs earlier [27].

### 4.1. Limitations

The main limitation of this study is the relatively small study cohort (resulting from a low number of NfL analyses). Another problem is the lack of dynamic follow-up of the NfL levels that could have provided a possible progression marker as well as their evaluation at the second time point. Since commercial sNfL level dosage was made available in Romania only in the past 3 years, funding and logistics did not allow us to use it for the T0 visit. Lack of a comparator group and lack of long term clinical follow-up are other weak points in our approach.

A possible bias comes from the fact that the majority of the patients in our study were treated with HETs for 1 to 3 years before evaluation. It is possible that the levels of sNfL were flattened as a result of therapy change before they entered the study. Longer follow-up and a study designed to clarify the effect of HETs on sNfL levels are needed.

### 4.2. Contribution to the Current Literature

Finally, our work aims to cover a gap in the current literature regarding the monitoring and treatment of MS, more precisely, the signification and importance of PIRA in patients that are still incorporated in RRMS groups. Whether discrete PIRA is typical for SPMS or whether it is a constant occurrence in RRMS is still unclear. The early detection of the transition from the stage of neuroinflammation (characteristic for the RRMS form) to the one in which neurodegeneration predominates (characteristic for the SPMS form) might be important because, as our study suggests, escalating therapy may not be sufficient to control the disease and prevent future incapacitation. To our knowledge, despite the increasing number of articles that address the issue of sNfLs in MS in recent years, there is still no study that could generate a consensus about the role of sNfLs in the progression of MS. In this direction, the results of the current study are yet another proof of the possible utility of detecting the sNfL level in MS patients, even in the case of those in whom other parameters suggest a “stationary” evolution.

With hope, we can say that these preliminary results could contribute to shape a new perspective in the long-term follow-up of MS patients and in their therapeutic approach, and we believe that the inclusion of sNfL detection in the clinical follow-up routine of these patients is a matter of a few years to come.

## 5. Conclusions

As the most worrying finding of our study, almost 80% of the RRMS patients (of whom 62.7% are treated with high-efficacy disease-modifying drugs) have shown some type of PIRA after 12 months, with no significant protective effect of the more aggressive treatment on progression. An EDSS event (increase with more than 1 point if initial EDSS was below 5 and with 0.5 points if initial EDSS was above 5.5) occurred in 24.1% of the patients, with a total of 30.1% experiencing any type of EDSS increase, above values reported for 12 months in most other studies. sNfL levels correlated with EDSS at both time points as well as with EDSS progression, but future research is necessary for a more accurate evaluation of their prognostic value. 

## Figures and Tables

**Table 1:** Clinical characteristics of the study group at T0 and T1.

Clinical Parameters (Average ± SD or Median and Q1, Q3)	T0	T1	Change
EDSS score	4 (2.5, 5.5)	4 (3.0; 6.0)	0 (0.0; 0.5)
9HPT time—dominant upper limb (s)	29.74 (23.61, 36.04)	30 (23.5, 37.0)	0.2% (−7.9, 9.08)
9HPT time—non-dominant upper limb (s)	31.66 (24.50, 36.74)	31.64 (24.15, 38.00)	0.94% (−6.06, 9.27)
9HPT time—highest change			2.07% (−8.67, 19.26)
25FWT average time (s)	6.67 (5.08, 10.64)	7.00 (4.75, 12.50)	6.84% (−4.76, 29.00)
SDMT (correct items)	32 (20, 41)	23 (16, 34)	−5 (−10, −2)
sNfL (pg/mL)		7.49 (5.44, 10.5)	−

EDSS: Expanded Disability Status Scale; 9HPT: 9-Hole Peg Test; 25FWT: 25-Foot Walk Test, SDMT: Symbol Digit Modalities Test.

**Table 2:** Proposed cutoff values for the tests used to diagnose SPMS, with sensitivity and 1-specificity values for the closest ROC determined cut-off values.

Test	Proposed Cut-Off Value to Diagnose SPMS (“SPMS Events”)	ROC Cut-Off Value	Sensitivity	1-Specificity
9HPT performance time increase	20%	20.135%	0.422	0.000
25FWT time increase	20%	20.261%	0.467	0.061
EDSS difference	1point/0.5points increase	1.25	0.156	0.000
SDMT score decrease	−4 items	−4.5	0.444	0.545
Disease duration	>10 years	10.5	0.200	0.438

9HPT: 9-Hole Peg Test, 25FWT: 25-Foot Walk Test; EDSS: Expanded Disability Status Scale; SDMT: Single Digit Modalities Test.

## Data Availability

Data are available upon request from the corresponding author.

## Supplementary Materials

The following supporting information can be downloaded at: https://www.mdpi.com/article/10.3390/medicina60091401/s1, Supplementary File S1 (statistics for correlations, logistic regression on SPMS event, ROC curves on SPMS event) and Supplementary File S2 (statistics for the influence of initial cognitive status, treatment duration on clinical parameters).
