# Supplementary material for: The Faces of “Too Late”—A Surprisingly Progressive Cohort of “Stable” Relapsing Remitting Multiple Sclerosis Patients
Source: Medicina (Kaunas). 2024 Aug 26;60(9):1401. doi: 10.3390/medicina60091401 (PMC11434352; doi:10.3390/medicina60091401)
Supplement: Supplementary file 1 [file medicina-60-01401-s001.zip › medicina-3154203-supplementary/suppl file 2.pdf]

Hypothesis Test Summary

	Null Hypothesis	Test	Sig.	Decision
1	The distribution of Nfl is the same across categories of Treatment type.	Independent-Samples Kruskal-Wallis Test	.236	Retain the null hypothesis.

Asymptotic significances are displayed. The significance level is .050.

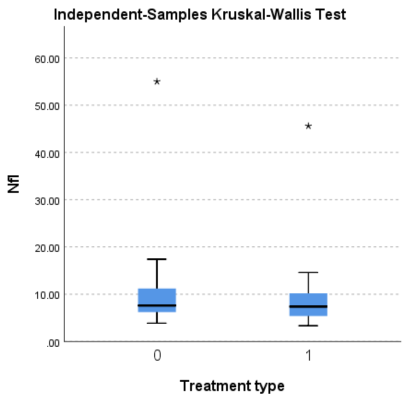

Independent Samples Kruskal-Wallis tests summary – no difference in sNfL levels in relation to the type of treatment (platform (0) versus HED (1))

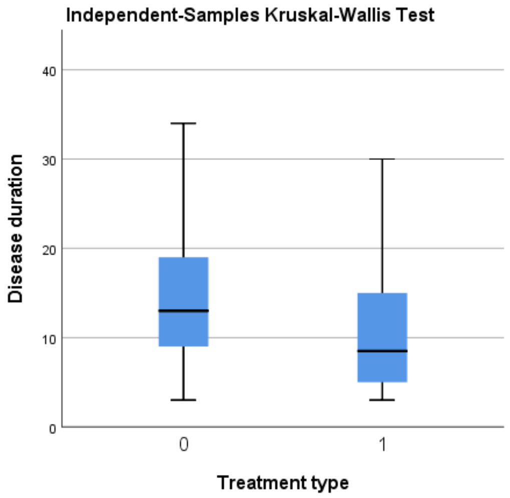

Independent-Samples Kruskal-Wallis Test Summary

Total N	83
Test Statistic	8.030[a,b]
Degree Of Freedom	1
Asymptotic Sig.(2-sided test)	.005

a. The test statistic is adjusted for ties.

b. Multiple comparisons are not performed because there are less than three test fields.

Disease duration is significantly higher for patients on platform therapy (0).

Hypothesis Test Summary

	Null Hypothesis	Test	Sig.	Decision
1	The distribution of SDMT difference is the same across categories of Treatment duration less than 3 years.	Independent-Samples Kruskal-Wallis Test	.286	Retain the null hypothesis.
2	The distribution of Nfl is the same across categories of Treatment duration less than 3 years.	Independent-Samples Kruskal-Wallis Test	.382	Retain the null hypothesis.
3	The distribution of 9HPT highest difference is the same across categories of Treatment duration less than 3 years.	Independent-Samples Kruskal-Wallis Test	.623	Retain the null hypothesis.
4	The distribution of EDSS difference is the same across categories of Treatment duration less than 3 years.	Independent-Samples Kruskal-Wallis Test	.751	Retain the null hypothesis.
5	The distribution of 25 FWT difference is the same across categories of Treatment duration less than 3 years.	Independent-Samples Kruskal-Wallis Test	.418	Retain the null hypothesis.

Asymptotic significances are displayed. The significance level is .050.

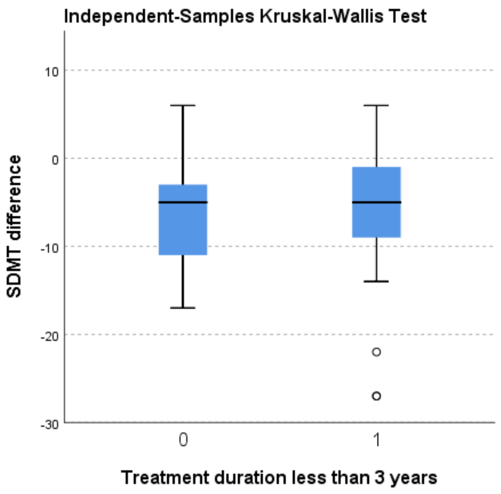

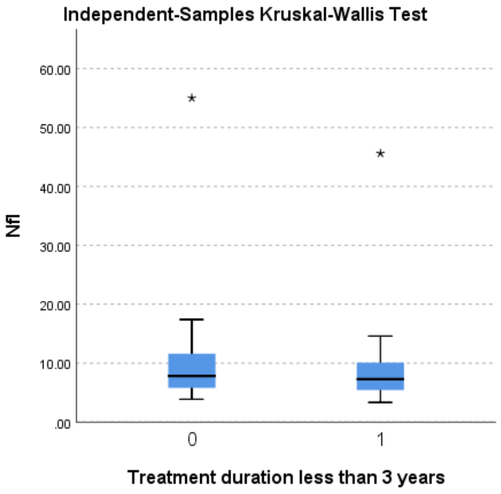

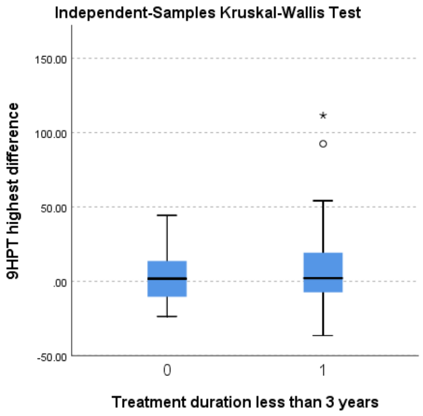

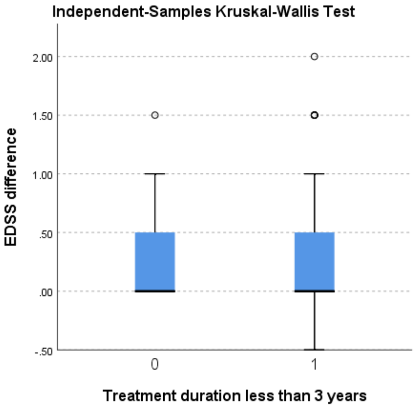

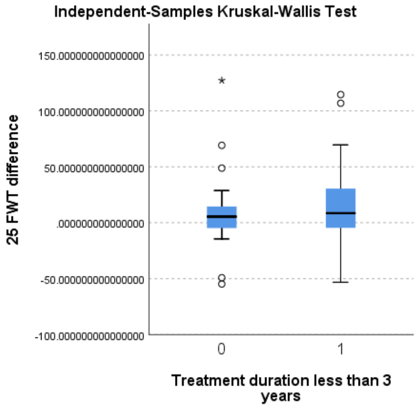

A current treatment duration of less than 3 years did not associate with differences in the evolution (differences) of EDSS, SDMT, 9HPT, 25 FWT and was not associated with signifficantly different levels of sNfL

Hypothesis Test Summary

	Null Hypothesis	Test	Sig.	Decision
1	The medians of SDMT difference are the same across categories of SDMT_normal.	Independent-Samples Median Test	.943[a]	Retain the null hypothesis.
2	The distribution of SDMT difference is the same across categories of SDMT_normal.	Independent-Samples Kruskal-Wallis Test	1.000	Retain the null hypothesis.
3	The medians of Nfl are the same across categories of SDMT_normal.	Independent-Samples Median Test	.846[a]	Retain the null hypothesis.
4	The distribution of Nfl is the same across categories of SDMT_normal.	Independent-Samples Kruskal-Wallis Test	.199	Retain the null hypothesis.

Asymptotic significances are displayed. The significance level is .050.

a. Yates's Continuity Corrected Asymptotic Sig.

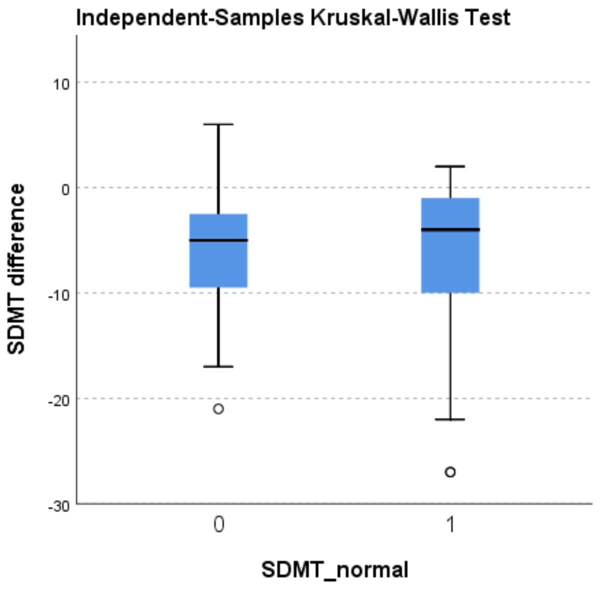

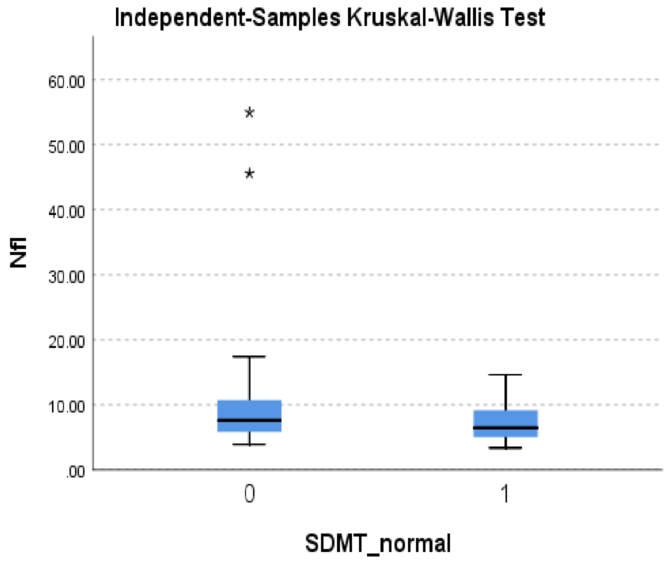

Normal cognitive state at T0 was not related to sNfL levels and did not influence cognitive loss (SDMT difference T1-T0)
